# Repetitive love proposing: A case report and review of phenomenology of impulsivity and compulsivity

**DOI:** 10.4103/0019-5545.37667

**Published:** 2007

**Authors:** Narayana Manjunatha, Devvarta Kumar, Haque S. Nizamie

**Affiliations:** Department of Psychiatry, Central Institute of Psychiatry, Ranchi, Jharkhand - 834 006, India; * Department of Clinical Psychology, Central Institute of Psychiatry, Ranchi, Jharkhand - 834 006, India

**Keywords:** Impulsive behavior, obsessive-compulsive disorder, proposing girls

## Abstract

Interest in opposite sex is a normal phenomenon; however, when this interest starts affecting one's own or others life in a pathological manner, it warrants clinical attention. We report the case of a young man who had a tendency to propose love to girls impulsively. Apart from the presence of this, otherwise, normal behavior in a pathological manner, another dimension of this case was that he was having obsessive-compulsive disorder too. Since both impulsivity and compulsivity are repetitive in nature and result in a sense of relief when the act is committed, the chance of impulsivity to be misconstrued as compulsivity is high. In light of important differences between compulsive and impulsive behavior, the psychopathology of the present case has been discussed.

Most of the patients with obsessive-compulsive disorder (OCD) present with the classical symptoms such as obsessions of dirt and obsessive doubts, leading to compulsive cleaning, checking and so forth. However, a range of other more unusual symptoms, such as compulsive joking and laughing, can be manifested by a few patients.[1,2] Moreover, patients can manifest symptoms that apparently present like obsession or compulsion but can be part of different diagnostic rubric. The unusual presentations can lead to diagnostic difficulty as well as delay in diagnosis. It is important for the clinicians to be aware of these unusual presentations to maximize appropriate diagnosis and early intervention.[3]

Among various co-morbidities of OCD, impulse control disorders (ICDs) are one of the commonest.[4] Impulsivity is defined as a predisposition toward rapid, unplanned reactions in response to internal or external stimuli without regard to consequences.[5] Compulsivity refers to repetitive behaviors performed according to certain rules or in a stereotyped fashion.[6] In diagnostic and statistical manual-IV (DSM-IV),[7] ICD is defined as the failure to resist an impulse, drive or temptation to commit an act that is harmful to the individual or to others. Furthermore, DSM-IV states that in most ICDs, the individual feels an increasing sense of tension or arousal before committing the act followed by feeling of pleasure, gratification or relief at the time of committing the act. In International Classification of Diseases-10 (ICD-10),[8] these conditions are classified as “habit and impulse disorders” and defined as repeated acts that have no clear rational motivation and, generally, harm the patient's own interests and those of other people.

Since there are various similarities between compulsivity and impulsivity, for example, both are repetitive in nature and provide a sense of relief, the chance of impulsivity to be misconstrued as compulsive behavior is high.[6] However, a clear understanding of these two phenomena is imperative from both the perspective of conceptualization of psychopathology and formulation of treatment strategy.[6]

Off late, spectrum concept in the context of OCD has started gaining impetus. Various disorders with commonality in terms of the presence of intrusive thoughts and repetitive behavior have been kept under the wider rubric of “obsessive-compulsive spectrum disorders” (OCSDs).[9] Apart from the presence of intrusive thoughts and repetitive behavior, these disorders can have similarities in terms of other features such as the age of onset, course, family history, neurobiological underpinning and response to treatment.[10] Moreover, it has been suggested that OCSDs fall on a spectrum between compulsivity and impulsivity.[9] The reason for conceptualization of compulsivity and impulsivity at two extreme is that firstly, there is an exaggerated sense of harm in compulsivity and underestimation of harm in impulsivity; and secondly, compulsive behavior is done in an attempt to decrease anxiety, whereas indulgence in impulsivity is due to the desire to obtain pleasure.[11] However, the pleasure-seeking aspect of impulsivity may not be generalizable across various subtypes of ICDs, e.g., patients of kleptomania may get pleasure from shop lifting, but the same may not be true for patients with trichotillomania who pulls up his hair.

We like to report the case of a 24-year-old male who presented with history of proposing love to girls as an impulsive phenomenon along with other repetitive behavior suggestive of OCD.

## CASE REPORT

A 24-year-old unmarried male, pursuing graduate studies hailing from higher socio-economic urban background with family history of one manic episode in a second-degree relative and nil significant past history, presented with 4 years illness of insidious onset, continuous and deteriorating course characterized by repetitive behavior of arranging things (pens, books, shoes, ties, clothes etc.) due to ideas of perfection. Initially, for about 1 year, even if these symptoms were causing impairment in socio-occupational functioning, the patient did not consider them irrelevant. However, gradually he started feeling that these thoughts and behavior were excessive, irrelevant, unwanted, intrusive and consuming a lot of time. Yet, initially he did not seek treatment. He used to feel distressed whenever he tried to resist these acts or reduce the duration of acts.

After 3 years of onset of illness, the patient started showing an unusual behavior of proposing love to girls impulsively. Whenever he would see a girl in any public place, he would have a strong desire to talk to her and propose her. Even though many times he felt that he should not repeat it, he could not control the impulse to talk and propose. Any hindrances in execution of this intention, for example, presence of own family members or the girl being involved in some other work would cause significant distress. Usually, he would approach the girl, introduce himself, praise her for physical characteristics and immediately express her liking to her. Most of the times, this sudden and unexpected way of proposing love would result in negative response from them; however, he would feel quite relaxed after doing so. In this manner, he proposed numerous girls in 1 year duration and many times, girls as well as authorities of his educational institute have complained his parents; subsequently, his classmate girls started avoiding him. Many a times, he missed his classes because of this behavior. When his parents asked the reason for proposing girls in this manner, his usual response was “*just like that*”. Many a times, he expressed to parents about his inability to control this behavior, parents, instead, blamed him of purposefully doing so. During interview, he expressed his inability to control the proposing behavior whenever he saw a girl and denied of any form of obsession associated with proposing behavior as well as had no intention of having sexual relationship or any plan of getting marriage. If due to any reason he was not able to propose girl, for example, if the girl left the place before he could approach her, he felt tensed and restless for a few minutes and then settled down.

His sleep, appetite, weight and libido were not disturbed. There was no history of repetitive washing or checking, any abnormal involuntary movements in any body parts, persistent elevated or depressed mood, increased psychomotor activity, substance abuse, delusions or hallucinations. Likewise, there was no history of stealing, fire setting, pulling hairs, excessive masturbation, skin picking and nail biting. As there was no temporal correlation between intake of anti-obsessive medicines and behavior of proposing girls, a chance of any hypomanic or manic switch, due to anti-obsessive medicine, was ruled out. This patient was diagnosed as having OCD with other habit and impulse disorders according to ICD-10.

Along with the behavior of proposing girls, various other repetitive behaviors in the form of arranging things were present. The patient and his family members felt about 40% improvements in his repetitive behavior, except the behavior of proposing girls, after around 8 weeks of treatment with 20 mg fluoxetine and 50 mg fluvoxamine. One month later in follow-up, carbamazepine-400 mg was added as anti-impulsive medication along with above medications, since there was no improvement in proposing behavior. In subsequent follow-up after 2 months, minimal improvement was seen in proposing behavior and about 60% improvement in other repetitive behavior and last follow-up subsequently.

## DISCUSSION

Presence of ICD in a patient with OCD is not uncommon. It is reported that 29% of OCD patients have co-morbid ICDs or other impulsive conditions.[4] However, this case report presents unusual presentation of ICD in a patient with OCD.

Interest in opposite sex is a normal phenomenon, which helps in establishing and maintaining a healthy sexual, marital and, in a broader perspective, interpersonal relationship. However, when this interest starts affecting one's own or others life in a pathological manner, it warrants clinical attention.[12] In the present case, the behavior of proposing girls was excessive in nature, did not have any clear motivation, was adversely affecting patient's day-to-day activities and his interactions with members of opposite sex. The behavior was repetitive in nature with failure to resist impulses and a sense of relief after carrying out the behavior. The diagnosis of “other habit and impulse disorders” according to ICD-10 and the “impulse control disorder not otherwise specified” according to DSM-IV were made based on these qualities. The possibility of these behaviors due to mood disorder (e.g., hypersexuality in patients with mania) was ruled out as there was no mood symptom at any point of time throughout the period of illness.

However, the question arises why this behavior is considered as impulsive phenomenon, more so, when there is a history of obsessions and compulsions in the same patient? Because, the impulsivity behaviors are mostly situational as specific situations provoke the individual to engage in impulsive behavior. Similarly, compulsions are “harm avoidance” behavior whereas impulsive behaviors are “risk taking” in nature.[3] Contrary to this, compulsions are the result of obsessions as patients engage in compulsive behaviors in order to decrease the distress and anxiety caused by the obsessive thoughts. In the present case, the behavior of arranging things was preceded by thoughts of perfection, whereas there was no indication for any obsession preceding the behavior of proposing girls and more so, these behaviors were situational specific, i.e., after seeing an isolated girl.

The comparison of phenomenology between impulsivity and compulsivity as well as their presence or absence in the reported case has been shown in Table 1.

**Table 1:** The comparison of phenomenology between impulsivity and compulsivity, as well as, their presence or absence in the reported case

No. Criteria	Compulsivity	Impulsivity	In the present patient	
			
			Behavior of arranging things (as compulsion)	Behavior of proposing girls (as impulsivity)
1. Repetitive behavior	Present	Present	Present	Present
2. Behavior being perceived as excessive	Present	Present	Present	Present
3. Behavior being perceived as irrational	Present	Absent	Present	Absent
4. Follows obsessive thought	Present	Absent	Present	Absent
5. Aimed at preventing or neutralizing anxiety	Present	Absent	Present	Absent
6. Performs with subjective sense of urge	Present	Present	Present	Present
7. Harm avoidance	Present	Absent	Present	Absent
8. Risk aversiveness	Present	Absent	Present	Absent
9. Risk seeking	Absent	Present	Absent	Present
10. Risk avoidance	Present	Absent	Absent	
			(He did not attach any sense of hazard with his arranging behavior)	Absent
11. Response to SSRI	Late but sustained response	Limited efficacy	Present	Absent

As far as OC symptoms in the patient are concerned, he had obsession of doing things perfectly, leading to arranging compulsion. A few commonly seen obsessions are obsessions of dirt and contamination and obsessive doubts. These obsessions lead to frequently noted compulsions such as compulsive cleaning, avoidance of, presumably, contaminated objects and checking compulsions. A relatively less frequently seen and less studied compulsion is compulsion to order and arrange one's surroundings and to ensure that objects are arranged in “exactly the right way”.[13] The present case has this obsession of perfection, leading to arranging compulsion.

However, the overlapping features (i.e., excessive, repetitive and performs with subjective sense of urge - criteria 1, 2 and 6 of Table 1) between compulsivity and impulsivity are considered by few authors as compulsive aspects of ICDs.[6]

There are many similarities between ICDs and bipolar mood disorder in terms of phenomenology (including harmful, dangerous or pleasurable behaviors, impulsivity and similar affective symptoms and dysregulation), age of onset (onset in adolescence or early adulthood), course, family history of mood disorder, possible central serotonergic and noradrenergic neurotransmitter abnormalities and response to mood stabilizers and antidepressants.[14] The present case has a few of these features. For example, the onset is in early adulthood and there is history of manic episode in a second-degree relative. However, in absence of core mood symptoms such as disturbance of mood, psychomotor activity and biological functions, the patient cannot be diagnosed of having affective illness.

To the best of our knowledge, this is the first case of proposing girls considered either as isolated ICD or as co-morbidity in a case of OCD, in published literature. It is also the first report in published literature, where ICD developed in later course of OCD. The report highlights two important issues firstly, how compulsivity and impulsivity look similar but there can be important phenomenological differences and secondly, how an age appropriate normal behavior can become pathological when it becomes excessive and starts disturbing the day-to-day activities of a person. Detailed assessment to differentiate compulsivity and impulsivity will guide to choose better interventions, either pharmacotherapy or behavior therapy and, in turn, improve the quality of life and reduce morbidity of patient.
